# Rapidly Manufactured CAR-T with Conserved Cell Stemness and Distinctive Cytokine-Secreting Profile Shows Improved Anti-Tumor Efficacy

**DOI:** 10.3390/vaccines12121348

**Published:** 2024-11-28

**Authors:** Shih-Ting Tsao, Mingyuan Gu, Qinghui Xiong, Yingzhi Deng, Tian Deng, Chengbing Fu, Zihao Zhao, Haoyu Zhang, Cuicui Liu, Xiong Zhong, Fang Xiang, Fei Huang, Haiying Wang

**Affiliations:** 1 Department of R&D, Shanghai HRAIN Biotechnology Co., Ltd., 1238 Zhangjiang Road, Pudong, Shanghai 201210, China; 2 Department of Regulatory Affairs, Shanghai HRAIN Biotechnology Co., Ltd., 1238 Zhangjiang Road, Pudong, Shanghai 201210, China; 3 Department of Medical Research, Shanghai HRAIN Biotechnology Co., Ltd., 1238 Zhangjiang Road, Pudong, Shanghai 201210, China

**Keywords:** chimeric antigen receptor T-cell, CD19, B-cell acute lymphoblastic leukemia, rapid manufacture, cytokine-secreting profile of CAR-T, stemness of CAR-T, anti-tumor efficacy of CAR-T

## Abstract

**Background:** The emergence of chimeric antigen receptor T-cell (CAR-T) immunotherapy holds great promise in treating hematologic malignancies. While advancements in CAR design have enhanced therapeutic efficacy, the time-consuming manufacturing process has not been improved in the commercial production of CAR-T cells. In this study, we developed a “DASH CAR-T” process to manufacture CAR-T cells in 72 h and found the excelling anti-tumor efficacy of DASH CAR-T cells over conventionally manufactured CAR-T cells. **Methods:** Four different CAR-T manufacturing processes were first proposed and examined by flow cytometry in regard to cell viability, T-cell purity and activation, CAR expression, and cell apoptosis. The selected two processes, 48H DASH CAR-T and 72H DASH CAR-T, were applied to the subsequent functional assessments, including T-cell differentiation, antigen-dependent cytotoxicity and expansion, cytokines secretion profile, and in vivo anti-tumor efficacy. **Results:** We demonstrated that rapidly manufactured CAR-T cells generated within 48–72 h was feasible and exhibited increased naïve and memory T-cell ratios, a distinctive secretory profile, superior expansion capacity, and enhanced in vitro and in vivo anti-tumor activity compared to conventionally manufactured CAR-T cells. **Conclusions:** Our findings suggest that “DASH CAR-T” process is a valuable platform in reducing CAR-T manufacturing time and producing high-efficacy CAR-T cells for future clinical application.

## 1. Introduction

B-cell acute lymphoblastic leukemia (B-ALL) stands as a malignancy characterized by the uncontrolled transformation and proliferation of lymphoid progenitor cells, impacting the bone marrow, blood, and extramedullary sites [1]. Chimeric antigen receptor-engineered T-cell (CAR-T) immunotherapy has emerged as a promising and effective approach for treating patients with B-ALL [2,3,4,5]. The high complete remission rates (CR), surpassing 90%, achieved by anti-CD19 CAR-T cell immunotherapy for recurrent/refractory B-cell acute lymphoblastic leukemia (R/R B-ALL) underscore its effectiveness [2,6].

The standardized manufacturing procedure for approved CAR-T cell therapy usually involves four sequential steps: (1) the collection of peripheral blood mononuclear cells (PBMCs) from enrolled patients; (2) the enrichment and activation of T-cells; (3) the transduction of T cells with CAR gene; and (4) the expansion and cryopreservation of CAR-T cells [7]. Despite the proven compatibility of this process in commercial manufacture, the considerable duration of manufacturing poses a significant obstacle as a rescue therapy for patients with progressing disease. Therefore, a rapid manufacturing procedure of CAR-T cells would greatly improve the accessibility and success of CAR-T therapy [3,4].

In this study, our primary focus was on addressing the challenge of lengthy CAR-T manufacturing times. Based on our previous experiences on the manufacture of CD19 autologous CAR-T, we have developed a novel manufacturing platform named DASH CAR-T. With rational design of compact manufacturing steps, we reduced the manufacturing time by concurrently activating and transducing T cells within a remarkable 48–72 h. Notably, our investigations revealed that CD19-targeted DASH CAR-T, with its distinctive secretory profile, exhibited superior expansion capability, higher ratios of naive and memory T cells, and enhanced anti-tumor activity compared to conventionally manufactured CAR-T cells.

## 2. Materials and Methods

### 2.1. CAR-T Cell Manufacturing

**Figure 1:**
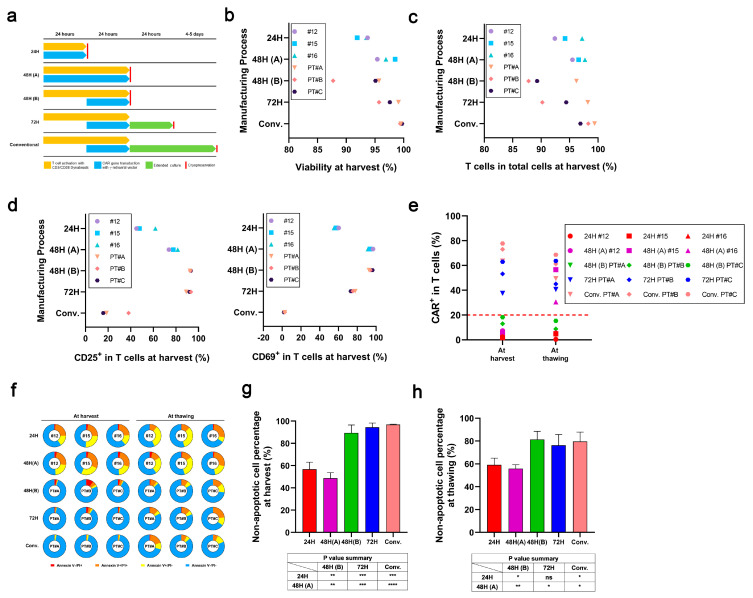
A novel approach to rapidly manufacture CAR-T cells. (**a**). Flow diagram for manufacturing processes of CAR-T. Three batches of healthy donors’ PBMCs (#12, #15, and #16) were used in 24 h and 48 h (A) processes and three batches of patients’ PBMCs (PT#A, PT#B, PT#C) were used in 48 h (B), 72 h, and Conv. CAR-T manufacturing processes. (**b**–**d**). Cell viability (**b**), T-cell proportion in total cells (**c**), and T-cell activation markers CD25 and CD69 expression (**d**) at harvest of CAR-T manufactured with different processes. (**e**,**f**). Percentage of CAR^+^ T cells in total cells (**e**) and apoptosis levels of CAR-T cells (**f**) of all five manufacturing processes at harvest and at thawing. (**g**,**h**). Significant differences of the proportion of non-apoptotic cells at CAR-T-cell harvest (**g**) and at CAR-T-cell thawing (**h**) between each manufacturing process. Data are plotted as mean ± SEM.

Peripheral blood mononuclear cells (PBMCs) were isolated using Sepax C-Pro Cell Processing System (Cytiva, Marlborough, MA, USA) from apheresis products obtained from consented patients enrolled in clinical trials (NCT03919526, NCT05651191, and NCT04303520) conducted by Hrain Biotechnology Co., Ltd. (Shanghai, China) T cells were isolated from PBMCs using CTS™ Dynabeads™ CD3/CD28 (40203D, ThermoFisher Scientific; Waltham, MA, USA). Activated T cells were transduced with γ-retroviral vector carrying the CD19 CAR sequence. The CAR virus was prepared in advance before the preparation of CAR-T. In detail, to generate the CD19 CAR constructs, the scFv domain of CD19 antibody FMC63 was cloned and fused to the CD8 hinge and transmembrane domain and intracellular domains derived from human CD28 and CD3ζ that was synthesized based on the genebank database and cloned into the 3rd generation retroviral vector MP71 using In-Fusion cloning (Takara, Otsu, Japan). Retroviruses were generated by first transfecting 70% confluent HEK 293vec-RD114 with transfer plasmid that was complexed with PEI at a DNA: PEI mass ratio of 1:3. For a confluent T25 flask, 3 μg of transfer plasmid was used for transfection. Media was changed 18 h after transfection and retroviral particles were harvested in the supernatant 48 and 72 h after transfection. The supernatant was then filtered through a 0.45 μm low protein binding filter and stored at −80 °C. For conventional CAR-T, the cells were kept in culture for four or five days after transduction without the presence of viral vectors. At the end of manufacture, CAR-T cells were harvested and cryopreserved. A graphic flowchart for each process (24 h, 48 h (A), 48 h (B), 72 h) is depicted in Figure 1a. The characteristics of the study participants are described in Table 1.

### 2.2. Flow Cytometric Analysis

Cells were co-incubated with fluorochrome-conjugated antibodies and the flow cytometry was performed on SA3800 Spectral Analyzer (SONY, Tokyo, Japan).

Antibodies used include the following: PerCP/Cyanine5.5 anti-human CD3 Antibody (1:100, BioLegend, 300328; San Diego, CA, USA); BV421 Mouse Anti-Human CD25 (1:200, BD Biosciences, 562442; Franklin Lakes, NJ, USA); Rabbit Anti-Mouse FMC63 scFv Polyclonal Antibody, Biotin (1:200, BioSwan, 500014; Shanghai, China); PE Streptavidin (1:200, BioSwan, 700032; Shanghai, China); FITC anti-human CD45RO (1:50, BioLegend, 304242; San Diego, CA, USA); and PE anti-human CD197 (CCR7) Antibody (1:100, BioLegend, 353204; San Diego, CA, USA).

### 2.3. Apoptosis Detection Assay

Annexin V-FITC Apoptosis Detection Kit (Beyotime, C1062; Shanghai, China) was used by following manufacturer’s instruction. The stained samples were examined by SA3800 Spectral Analyzer (SONY).

### 2.4. Cell Cycle Assay

Cell Cycle Detection Kit (abs50005, Absin; Shanghai, China) was used according to manufacturer’s instruction to perform the cell cycle examination. The fluorescence was detected by SA3800 Spectral Analyzer (SONY).

### 2.5. Cytotoxicity Assay

The ability of CAR-T cells to kill CD19^+^ target cells was evaluated using Bright-Glo™ Luciferase Assay System (E2620, Promega; Madison, WI, USA). The target cells were CD19^+^ Nalm6 target cells engineered to stably express Luciferase-GFP (LUC-GFP). CAR-T cells or non-transduced T (NT) cells were co-cultured with target cells at a 1:5 E:T ratio in 96-well plates for 24–120 h. The co-culture system was sampled every 24 h and tested following manufacturer’s instruction. Luminescence was detected using a plate reader (VICTOR^®^ Nivo™ Multimode Plate Readers, PerkinElmer, Yokohama, Japan). The percentage of perished Nalm6 were calculated as (1 − readings of co-culture group/readings of target-cell-only group) × 100%.

### 2.6. Antigen-Dependent Expansion of CAR-T Cells

CAR-T cells were co-cultured with CD19^+^ Nalm6-LUC-GFP or CD19 knock-out (KO) Nalm6-LUC-GFP target cells at a 1:1 E:T ratio for 11 days. The co-culture system was sampled every two to three days. The sampled cells were applied to flow cytometry for cell counting with CountBright™ Plus Absolute Counting Beads (ThermoFisher Scientific, C36995; Waltham, MA, USA) for CAR^+^ cell analysis with above mentioned anti-FMC63 antibody and for GFP^+^ cell proportion analysis. CAR-T cells were identified as CAR^+^GFP^−^ cells and the expansion fold was calculated based on the CAR^+^ T cell number at the beginning of co-culture.

### 2.7. Cytometric Bead Array (CBA)

Culture supernatant was then collected from cytotoxicity assay and examined with Cytometric Bead Array (CBA) Human Th1/Th2/Th17 Cytokine Kit (560484, BD Biosciences; Franklin Lakes, NJ, USA) according to the instruction manual.

### 2.8. Luminex-Based Cytokine/Chemokines Assay

CAR-T cells and NT cells were co-cultured with either CD19^+^ Nalm6-LUC-GFP target cells or CD19 knock-out (KO) Nalm6-LUC-GFP target cells at E:T ratios of 1:5, 1:20, and 1:40. Supernatant was collected after 24 h of co-culture and examined by Bio-Plex Pro Human Cytokine Screening Panel, 48-Plex (12007283, Bio-Rad; Hercules, CA, USA) and Luminex 200 system (Luminex Corporation, Austin, TX, USA). The concentration of cytokines was calculated based on fluorescence intensity and standard curves.

### 2.9. In Vivo Models

We used 6–8 weeks old immunodeficient NOG mice inoculated with luciferase-GFP-expressing Nalm6 cells (human pre-B ALL cell line) to assess the anti-tumor activity of CAR-T in vivo. The mice were housed in cages and maintained on a 12 h light/dark cycle with access to food and water ad libitum. NOG mice were intravenously injected with 5E5 luciferase-GFP-expressing Nalm6 cells three to four days before CAR-T infusion. On the infusion day, 100 tumor-bearing mice were randomly assigned to treatment/control groups (five mice/group) according to body weight and intravenously injected with CAR-T cells or NT cells manufactured with PBMCs from a healthy donor a B-ALL patient at different doses (conventional CAR-T: 1E6 and 5E6 CAR^+^ T cells per mouse; 48H DASH CAR-T and 72H DASH CAR-T: 2.5E5, 5E5, and 1E6 CAR^+^ T cells per mouse; NT: total T cell number as in conventional 5E6 group), or 200 μL vehicle (homemade cryopreservation solution for CAR-T cells). Tumor growth was monitored weekly by IVIS Spectrum system performing bioluminescent imaging. Peripheral blood was sampled weekly, and flow cytometry was performed to evaluate the existence of CAR-T cells. Kaplan–Meier survival data were analyzed by the log-rank (Mantel–Cox) test. All mice were included in the study if they underwent successful tumor inoculation. The mice were excluded from subsequent studies if the mice died.

### 2.10. Statistical Analysis

Statistical analysis was conducted using GraphPad Prism software (version number: 8.0.1). The graphs represent the mean value ± SEM, unless otherwise indicated. Statistics of proportion of non-apoptotic cells were conducted using un-paired t-test when appropriate. *p*-values < 0.05 were considered statistically significant.

## 3. Results

### 3.1. CD19 DASH CAR-T Shortened Manufacture Time to 48–72 h

We first designed four rapid CAR-T manufacturing procedures, noted as 24 h, 48 h (A), 48 h (B), and 72 h (Figure 1a). In order to determine the optimal CAR-T manufacture, we evaluated parameters including cell viability (Figure 1b), T-cell % (Figure 1c), T cell activation (Figure 1d), CAR^+^ expression (Figure 1e), and apoptosis (Figure 1f–h) during the manufacturing process and after CAR-T-cell thawing. There was no significant difference in cell viability, T-cell proportion, or T-cell activation markers CD25 and CD69 expression among all processes (Figure 1b–d). However, we found that little CAR^+^ expression was detected in the 24 h CAR-T at harvest and at different timepoints in the culture after thawing (Figure 1e). In addition, increased apoptosis with a concurrently significant reduced proportion of non-apoptotic cells was observed in 24 h and 48 h (A) CAR-T compared with 48 h (B) and 72 h or conventional CAR-T both at harvest and at thawing (Figure 1f–h). Therefore, the 48 h (B) and 72 h processes were capable of producing viable CAR-T cells and formed the DASH CAR-T platform.

### 3.2. DASH CAR-T Possesses Higher Stemness, Improved Expansion Ability, Greater Cytotoxicity, and Enhanced Cytokines Release Against Target Cells

**Figure 2:**
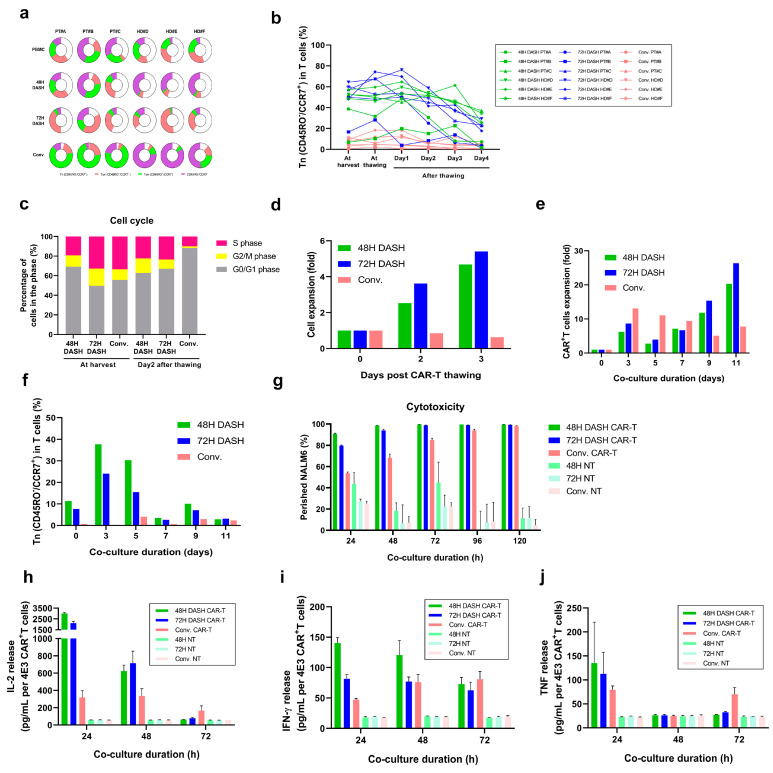
DASH CAR-T exhibited higher stemness, greater cytotoxicity, and enhanced expansion ability in vitro. (**a**). The proportion of T-cell subtypes classified with CD45RO/CCR7 expression in PBMCs at CAR-T harvest. T cell subtypes including Tn (CD45RO^−^/CCR7^+^), Tcm (CD45RO^+^/CCR7^+^), and Tem (CD45RO^+^/CCR7^−^). HD: healthy donor. (**b**). Changes of proportion of Tn (CD45RO^−^/CCR7^+^) in CAR-T cells at harvest and after thawing. (**c**). Proportion of cells at different phases of cell cycle of CAR-T cells at harvest and after thawing. (**d**,**e**). Expansion of CAR-T cells after thawing (**d**) and during co-culture with CD19^+^ Nalm6 target cells (**e**,**f**). Changes in proportion of Tn (CD45RO^−^/CCR7^+^) in CAR-T cells during co-culture with CD19^+^ Nalm6 target cells. (**g**). Cytotoxicity of CAR-T cells on CD19^+^ Nalm6 target cells at different co-culture durations. (**h**–**j**). IL-2 release (**h**), IFN-γ release (**i**), and TNF release (**j**) in the supernatant of co-culture of CAR-T or NT cells with CD19^+^ Nalm6 target cells for 24 h, 48 h, and 72 h.

To investigate whether DASH CAR-T is more potent in eliminating target cells compared to conventional CAR-T, we performed an in vitro functional assessment. Compared to conventional CAR-T, DASH CAR-T exhibited a less-differentiated phenotype (Tn and Tcm) at harvest, which is more similar to the phenotype of starting material PBMCs (Figure 2a). After thawing, a high proportion of Tn cells were maintained in healthy donor DASH CAR-T and patient (PT) DASH CAR-T, with only slight decline at Day 3 and Day 4 (a more obvious decline was observed only in PT#A starting from Day 2), while the proportion of Tn cells in conventional CAR-T remained low for all donors (Figure 2b). In cell cycle analysis, more DASH CAR-T were in a proliferative (G2/M) state, with an obviously higher proportion of G2/M phase relative to conventional CAR-T, especially on Day 2 after thawing (Figure 2c). The observation was supported by the continuing expansion of DASH CAR-T cells after thawing with no antigen presence (Figure 2d).

In vitro anti-tumor functionality was evaluated through antigen-dependent CAR-T expansion assay, cytotoxicity assay, and cytokine bead array. Although DASH CAR-T showed a lower expansion ability in the early stage after exposure to CD19^+^ Nalm6 cells compared to conventional CAR-T, its persistent proliferation gradually exceeded that of conventional CAR-T and the expansion folds were even elevated after Day 9 co-culture (Figure 2e). In addition, DASH CAR-T kept a higher proportion of the Tn cell population than conventional CAR-T at any time points in the co-culture system (Figure 2f). DASH CAR-T showed significantly greater cytotoxicity against Nalm6 tumor cells compared to conventional CAR-T in 24 h, 48 h, 72 h, and 96 h co-culture (Figure 2g). The data also suggested a fast onset of the antigen-dependent cytotoxicity of DASH CAR-T cells. Culture supernatant was collected from the cytotoxicity assay and the cytokine release was measured by CBA. Under the exposure of CD19^+^ Nalm6 cells for 24 and 48 h, significantly enhanced production of IL-2 (Figure 2h) and IFN-γ (Figure 2i) and an elevation trend of TNF (Figure 2j) secreted by DASH CAR-T was observed. According to these results, DASH CAR-T outperformed conventional CAR-T in anti-tumor efficacy in vitro.

### 3.3. CD19 DASH CAR-T Showed Distinctive Cytokines Secretion Profile

**Figure 3:**
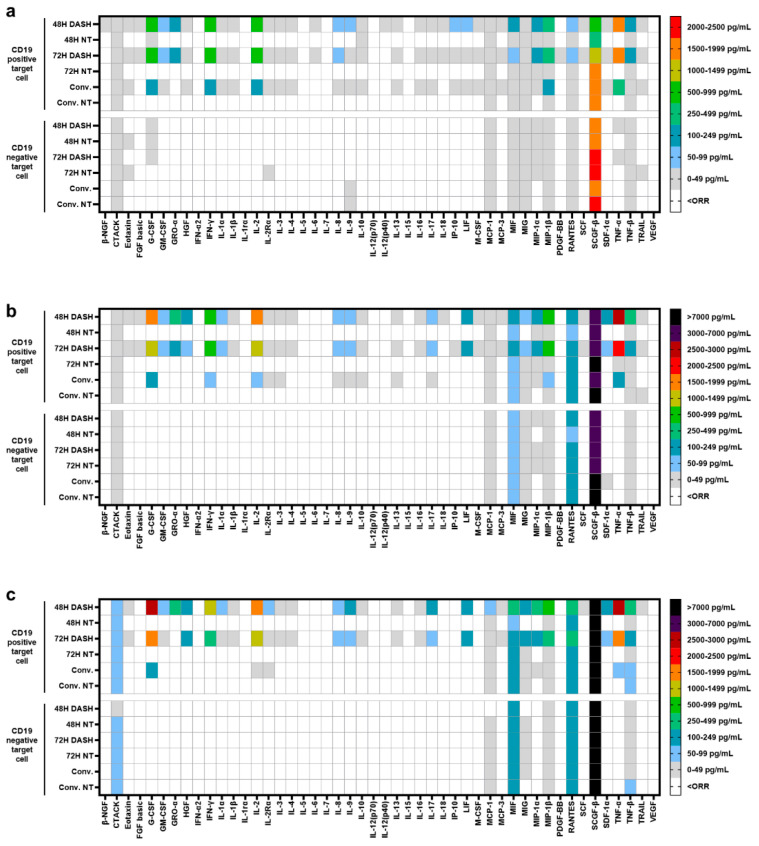
Cytokine/chemokine secretion profile of 48 h DASH CAR-T, 72 h DASH CAR-T, and conventional CAR-T. Heat map analysis of cytokines/chemokines secreted in the supernatant of CAR-T cells or NT cells co-cultured with CD19^+^ Nalm6 cells or CD19 KO Nalm6 cells for 24 h at a 1:5 E:T ratio (**a**), 1:20 E:T ratio (**b**), and 1:40 E:T ratio (**c**).

Since T-cell-secreted cytokines are associated with the expansion, persistence, and anti-tumor efficacy of CAR-T cells, we investigate deeper into the cytokine-releasing profile of DASH CAR-T cells and conventional CAR-T cells with Luminex-based cytokine/chemokines assay. As shown in Figure 3, DASH CAR-T exhibited an enhanced antigen-specific cytokine secretion. DASH CAR-T produced significantly higher levels of G-CSF, IFN-γ, IL-2, and TNF-α (>1000 pg/mL) than conventional CAR-T when co-cultured with CD19 positive target cells (Figure 3a–c), which was consistent with our previous cytokine assay results (Figure 2h–j). Moreover, the difference in cytokine secretion between DASH CAR-T and conventional CAR-T was more pronounced at low E:T ratios (Figure 3b,c). We also observed that DASH CAR-T produced slightly higher levels of MIP-1α, MIP-1β, and TNF-β than conventional CAR-T. Overall low levels of cytokines, except SCGF-β, were detected in DASH CAR-T and conventional CAR-T co-cultured with CD19-negative target cells, as well as in NT cells co-cultured with CD19 positive/negative target cells.

### 3.4. CD19 DASH CAR-T Cells Demonstrated Enhanced Anti-Tumor Efficacy and Increased Expansion and Persistence In Vivo

**Figure 4:**
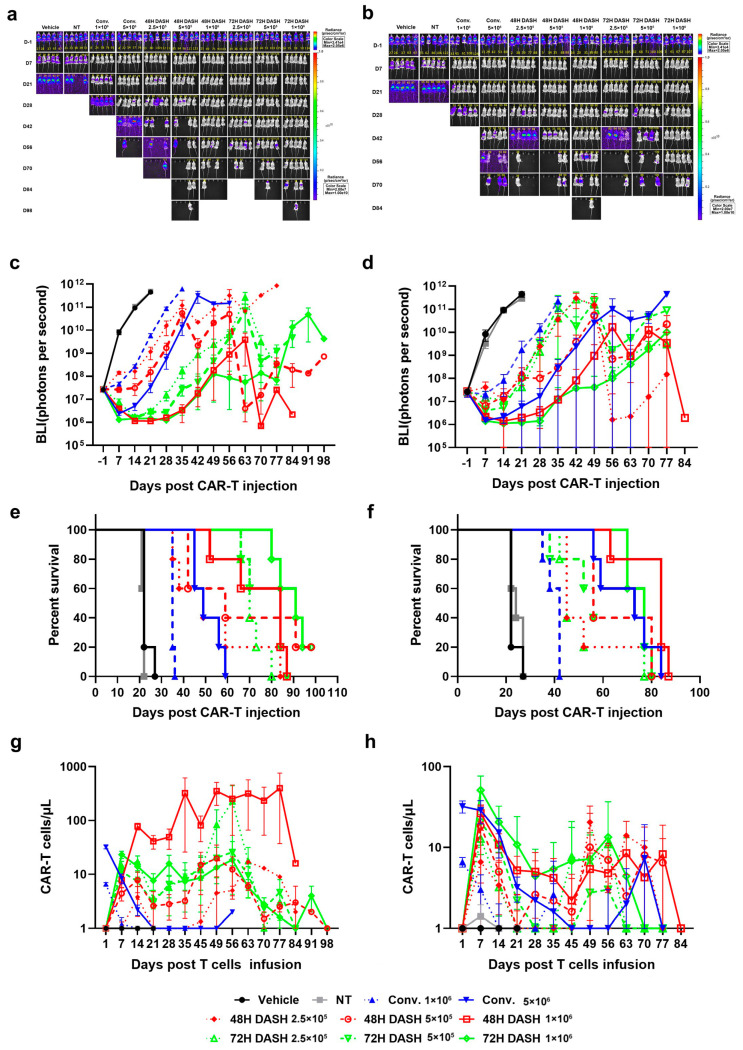
DASH CAR-T showed superior anti-tumor efficacy and CAR-T expansion compared to conventional CAR-T in vivo. (**a**,**b**). Images of tumor-bearing NOG mice injected with CAR-T cells manufactured with PBMCs from a healthy donor (**a**) or a B-ALL patient (**b**). Mice were injected with different T-cell doses. Conventional CAR-T: 1E6 and 5E6 CAR^+^ T cells per mouse; 48 h DASH CAR-T and 72 h DASH CAR-T: 2.5E5, 5E5, and 1E6 CAR^+^ T cells per mouse; NT: total T-cell number as in conventional 5E6 group. (**c**,**d**). Bioluminescence intensity of tumor-bearing NOG mice injected with CAR-T cells manufactured with PBMCs from a healthy donor (**c**) or a B-ALL patient (**d**). (**e**,**f**). Survival curves of tumor-bearing NOG mice injected with CAR-T cells manufactured with PBMCs from a healthy donor (**e**) or a B-ALL patient (**f**). (**g**,**h**). CAR-T expansion in peripheral blood of tumor-bearing NOG mice injected with CAR-T cells manufactured with PBMCs from a healthy donor (**g**) or a B-ALL patient (**h**).

We further evaluated the anti-tumor efficacy of DASH CAR-T and conventional CAR-T in vivo. After CAR-T infusion, both DASH CAR-T and conventional CAR-T manufactured with PBMCs from a healthy donor (Figure 4a,c) or a B-ALL patient (Figure 4b,d) could eradicate tumor. However, between the same dosage groups (Conv. 1E6, 48 h DASH 1E6, and 72 h DASH 1E6), the relapse of tumor was seen in the Conv. 1E6 group in the third week and the DASH CAR-T groups remained tumor-free up to 2 months. Mice infused with DASH CAR-T achieved a noticeably prolonged survival compared to mice infused with conventional CAR-T. Seventy-two days after CAR-T injection, only mice in the 72 h DASH 1E6 groups with CAR-T derived from a healthy donor maintained 100% survival (Figure 4e). Similarly, at 56 days after CAR-T injection, only mice in the 72 h DASH 1E6 groups with CAR-T derived from a B-ALL patient maintained 100% survival (Figure 4f). In contrast, mice treated with 1E6 conventional CAR-T derived from a healthy donor or a B-ALL patient died at 28 days after CAR-T injection. DASH CAR-T displayed a more persistent anti-tumor capacity and resulted in overall survival benefit over conventional CAR-T.

CAR-T expansion was analyzed by sampling the peripheral blood of mice at different time points after CAR-T injection. The CAR^+^ T cell counts per µL in the peripheral blood were calculated by flow cytometry. A considerable increase in and the enhanced persistence of CAR^+^ T cells was observed in mice injected with DASH CAR-T compared to conventional CAR-T, regardless of whether the CAR-T cells were produced from a healthy donor (Figure 4g) or a B-ALL patient (Figure 4h). In addition, CAR^+^ T cells were undetectable in mice injected with conventional CAR-T produced from healthy donor after Day 21 post infusion while CAR^+^ T cells stayed almost 1000 cells per µL blood in the mice in the DASH 48 h 1E6 groups.

Based on the results established herein, we have developed a rapid CAR-T manufacturing strategy that generated less differentiated CAR-T cells with a distinctive cytokine secretion profile and improved cytotoxic and expansion ability.

## 4. Discussion

In the study, we proposed a novel approach named DASH CAR-T to rapidly generate CAR-T cells and present in vitro and in vivo evidence that DASH CAR-T cells displayed higher stemness, improved cytotoxic and expansion ability, distinctive secreted cytokine profile, and enhanced anti-tumor efficacy compared to conventionally manufactured CAR-T cells.

The result from the first part of the study, which focused on process development, implicated that CAR gene could be transduced into activated T cells and render the expression of functional CAR protein in 48 to 72 h using γ-retroviral vector. According to our findings, the expression of CAR on T cells in CAR-T manufactured in 24 h (process “24 h”) was barely detected at harvest and even in prolonged culture after thawing. In addition, the result suggested that the sufficient activation of T cells was crucial to the viability of CAR-T cells, as revealed by the poor non-apoptotic cell proportion at harvest of process 24 h and 48 h (A), in which T-cell activation and viral transduction started simultaneously.

The second part of the study emphasized the unique characteristics of DASH CAR-T in the aspects of cell differentiation status, cell cycle stage, cytotoxicity, and cytokine release. Studies have shown that the prolonged manufacturing time of CAR-T cells may cause accelerated cell differentiation with less naive-like (Tn: T naïve cell) and central memory cells [8,9,10,11,12]. Less differentiated T cells lead to higher expansion, longer persistence, and enhanced anti-tumor efficacy in vivo. Clinical studies also found that the transcriptome of CAR-T cells in complete-responding patients were enriched with the memory-related gene while CAR-T cell differentiation signaling pathways in non-responding patients were up-regulated [10,11,13,14,15,16]. T naïve cells, in particular, expand and persist in vivo for a significant period of time and improve the clinical outcome [15,17,18,19]. These findings were reaffirmed in our results of in vitro and in vivo functional evaluation. We found that DASH CAR-T maintained the less-differentiated phenotype at harvest similar to PBMCs and possessed a high proportion of Tn cells after thawing or co-culture with Nalm6 target cells in vitro. DASH CAR-T also showed superior anti-tumor efficacy and persistent CAR-T cells expansion in the in vivo tumor model. This result highlights that DASH CAR-T cells outstand conventional CAR-T cells with their remarkable tumor-killing ability.

Besides the differentiation status and the expansion ability of CAR-T, antigen-dependent cytokine release also plays a crucial role for therapeutic success. In our study, we found that antigen-stimulated DASH CAR-T secreted more abundant IL-2, IFN-γ, TNF, MIP-1α, MIP-1β, and G-CSF than conventional CAR-T. IL-2 is a crucial T-cell cytokine that promotes T-cell proliferation and effector function, and is considered as an effective immunotherapy treatment for cancer [20,21,22,23]. It is plausible that the enhanced expansion ability and persistence of DASH CAR-T cells under the exposure of in vitro and in vivo antigen benefit from their higher levels of IL-2 secretion. IFN-γ has been an index in the functional evaluation of CAR-T products and its increased secretion is generally concurrent with an enhanced anti-tumor efficacy [10,24,25]. TNF-α and TNF-β are two representative members of TNF superfamily with similar activity and can exert cytotoxic effects [26], and TNF-α, especially, is an important anti-tumor cytokine in addition to IFN-γ [10,27,28]. Our results revealed that DASH CAR-T, which secreted higher levels of IFN-γ and TNF immediately after antigen presentation compared to conventional CAR-T, exhibited greater tumor-eradication ability in vitro and in vivo. MIP-1α (CCL3) and MIP-1β (CCL4) are highly related members of the CC chemokine subfamily [29]. MIP-1α and MIP-1β act as T-cell chemokines that facilitate the recruitment of effector T cells to the tumor site and drive the priming of effective anti-tumor immunity [30,31,32,33]. It was speculated that the modest elevation of MIP-1 and MIP-1β secreted by DASH CAR-T may also contribute to enhancing the anti-tumor efficacy. G-CSF is often used to mitigate neutropenia after CAR-T infusion. However, the relationship between G-CSF administration and the toxicity and efficacy of CAR-T-cell therapy is unclear. Recent research demonstrated that Early G-CSF administration significantly decreased the incidence of febrile neutropenia, without any impact on toxicities or the anti-tumor efficacy of CAR-T [34,35]. This suggested that DASH CAR-T with robust G-CSF release may alleviate the associated toxicities of CAR-T by overcoming severe hemocytopenia. However, more studies are needed to prove this hypothesis. On the other hand, low-level IL-6 secretion from DASH CAR-T may indicate a better safety of the cells in the aspect of inducing cytokine release syndrome [36].

Intriguingly, a substantially high release of SCGF-β was observed in in every co-culture system. Although newly discovered, the elevated level of SCGF-β had been reported in several cancer types. Leukemic cells also required self-secreted SCGF for their proliferation [37,38]. However, little is known about the possible role played by SCGF-β in CAR-T immunotherapy and more research on this observation is expected.

In our study, we used PBMCs collected from both B-ALL patients and healthy donors to produce CAR-T cells. Patient PBMC might increase the risk of manufacturing failure, a reduction in Tn cell proportion, or even the poor anti-tumor efficacy of CAR-T cells in vivo [39,40]. Nevertheless, DASH CAR-T were successfully manufactured from B-ALL patients’ PBMC and demonstrated a function comparable to that of healthy donor CAR-T in vitro and in vivo. The result strongly supports the clinical potential of DASH CAR-T. Furthermore, although the in vivo study demonstrated that DASH CAR-T prepared from a patient with B-ALL or a healthy donor exhibited exceptional anti-tumor efficacy at a dose lower than that of conventional CAR-T. However, at the same dose, there are minor disparities in the treatment outcomes between DASH CAR-T from a patient and that from a healthy donor. Dose factors play a crucial role in diverse illness areas and drug therapies. Thus, when determining drug doses for clinical administration, numerous aspects must be taken into account, including patient characteristics, diseases severity, drug action mechanisms, and pharmacokinetic properties [41,42,43,44].

In summary, we have developed a novel and time-saving manufacturing strategy, DASH CAR-T, to generate CAR-T cells with γ-retroviral gene delivery system. We demonstrate that CAR-T cells manufactured through the DASH CAR-T process possess improved functionality in vitro and in vivo, evidenced by less terminal differentiation, higher cytotoxicity, and prolonged persistence. This outstanding anti-tumor efficacy of DASH CAR-T cells is related to their distinctive cytokines-secreting profiles that differ from those of conventional CAR-T cells. Based on the results of this preclinical study, we anticipate that the DASH CAR-T process be used in the manufacture of CAR-T cells in clinical application and promote accessibility to CAR-T cell immunotherapies for patients in need.

## Figures and Tables

**Table 1:** The characteristics of the study participants.

Number	Individual Identification Number	Disease Status	Gender	Age/Years	Weight/kg
#12	SC12079	Healthy	Male	21	65
#15	SC12305	Healthy	Male	32	57
#16	SC12021	Healthy	Male	21	65
#A	PAYF-12-003	B-ALL patient	Female	56	55
#B	LISH-05-031	B-ALL patient	Female	52	61
#C	ZHJP-09-007	B-ALL patient	Male	63	55

## Data Availability

The datasets generated and analyzed during the current study are not publicly available due to the confidentiality agreement of Hrain Biotechnology Co., Ltd. Still, they are available from the corresponding author on reasonable request.

## List of Abbreviations

CAR-T: chimeric antigen receptor T-cell; B-ALL: B-cell acute lymphoblastic leukemia; IL-2: interleukin 2; PBMCs: peripheral blood mononuclear cells; NT cell: non-transduced cell; IFN-γ: interferon gamma; TNF: tumor necrosis factor; IL-6: interleukin 6; G-CSF: granulocyte colony-stimulating factor; SCGF-β: stem cell growth factor β; PT: patient; KO: knockout; Tn: naïve T cell; Tcm: central memory T cell; Tem: effector memory T cell; Conv. CAR-T: conventional CAR-T; DASH CAR-T: rapidly manufactured CAR-T.
